# Effect of Altered Cervical Thread Pitch on the Primary Stability of Dental Implants

**DOI:** 10.3390/jcm15020864

**Published:** 2026-01-21

**Authors:** Lászlo Major, Ibrahim Barrak, Gábor Braunitzer, József Piffkó, Mark Adam Antal

**Affiliations:** 1 Department of Oral and Maxillofacial Surgery, Faculty of Medicine, University of Szeged, 6720 Szeged, Hungarypiffko.jozsef@med.u-szeged.hu (J.P.); 2 dicomLAB Dental Ltd., 6720 Szeged, Hungary; 3 Department of Operative and Esthetic Dentistry, Faculty of Dentistry, University of Szeged, 6720 Szeged, Hungary

**Keywords:** dental implants, insertion torque, osteotomy, periotest, resonance frequency analysis

## Abstract

**Background:** The macrogeometry and shape of dental implants strongly influence primary stability, which may at times result in excessively high insertion torque. This in vitro study aimed to evaluate whether increasing coronal thread density could reduce insertion torque without compromising primary stability. **Methods:** Two conical implants with identical macrogeometry and surface characteristics (Ø 4.2 × 11.5 mm) differed only in the thread pitch of the coronal 3 mm: a modified version (27% more coronal threads; Group 1) and a standard, commercially available version (Group 2). Thirty implants of each design were inserted into high-density (D1; 40 PCF; pounds per cubic foot) and low-density (D3; 20 PCF) polyurethane blocks (n = 120). Insertion torque (IT) and implant stability quotient (ISQ, measured by resonance frequency analysis) were recorded. Group comparisons used the Kruskal–Wallis test, and a generalized linear model (GLM) assessed the independent effects of IT and design on ISQ in D1 bone. **Results:** In D1 bone, Group 2 showed higher IT (median 74.0 vs. 63.5 N·cm; *p* < 0.001) and ISQ (mean 79.1 vs. 77.4; *p* ≤ 0.030). The GLM identified IT as a negative predictor of ISQ (β = −0.267 per 1 N·cm; *p* < 0.001), and Group 2 was associated with higher ISQ (+3.90; *p* < 0.001). In D3 bone, Group 2 again exhibited higher IT (median 37.5 vs. 33.0 N·cm; *p* < 0.001), while ISQ values were similar between designs (all *p* > 0.35). **Conclusions:** Increasing coronal thread density lowers insertion torque without reducing stability in softer bone and maintains sufficient ISQ for immediate loading in dense bone, making the design advantageous for varied bone qualities.

## 1. Introduction

Today, dental implants are considered a reliable and successful treatment modality for restoring partial or full edentulism in oral surgery and dental medicine. Long-term studies have reported survival rates exceeding 95% [1,2,3,4,5]. Since the 1980s, implant surface modifications have been shown to enhance osseointegration by promoting osteophilic surfaces that increase bone-to-implant contact and accelerate bone formation, thereby improving early implant stability [6,7].

Researchers have enhanced implant performance through various surface modification techniques, including physical, chemical, electrochemical, deposition, and biochemical methods [8,9]. Many of these investigations have been carried out on implants with various macro-designs, diameters, lengths, thread spacing and depth, micro-threads, and other geometrical features of dental implants [10,11,12].

Recent evidence indicates that implant thread pitch is a key macro-design variable influencing insertion torque and primary stability, with altered pitch showing measurable changes in stability outcomes [13]. At the same time, clinical data show insertion torque and ISQ are related but not equivalent metrics, meaning higher torque does not always translate linearly into higher stability [14]. However, most available studies vary pitch along the whole implant or compare global thread designs, leaving a specific gap: the independent biomechanical contribution of coronal thread pitch (crestal region) to the torque–stability trade-off remains insufficiently isolated and quantified [15]. Primary stability is defined as the biomechanical stability measured immediately after implant insertion; it reflects the extent of bone–implant contact (BIC) [16] and results from the mechanical engagement of the implant with the surrounding bone. It is considered a key determinant of long-term implant stability and success. Primary stability improves as micromovements between the implant and surrounding bone decrease, enabling faster and more reliable healing and osseointegration [17,18].

Implant stability immediately after insertion and the absence of micromovements are likewise critical in determining whether immediate loading is feasible. Adequate fixation prevents the formation of connective tissue at the bone–implant interface. It is well documented that certain levels of micromotion can damage blood vessels and cells. Micromotion between the implant and surrounding bone should not exceed a threshold of 150 µm for successful osseointegration; movements above this threshold may induce stress, impair cell recruitment, and negatively affect bone remodeling and healing, leading to the formation of fibrous tissue [17,18,19,20].

Different techniques are available to assess dental implant stability. Among these, resonance frequency analysis (RFA) using the Osstell device (Osstell AB, Gothenburg, Sweden) is widely employed. This method measures the oscillation frequency of the implant within the bone, induced by a magnetic pulse, and expresses it as an implant stability quotient (ISQ) on a scale from 1 to 100 [21,22,23]. In vitro studies have shown that ISQ values increase with the stiffness of the bone–implant interface. RFA is therefore a useful clinical tool for monitoring the progression of osseointegration, as it allows assessment of both primary stability (immediately after insertion) and secondary stability achieved during the subsequent healing period [21]. Insertion torque (IT), measured with a torque meter at placement, provides complementary information regarding local bone density and, to some extent, implant stability; higher IT values typically indicate reduced micromotion during the early healing phase, before osseointegration occurs [21,23].

From another perspective, achieving “high mechanical implant stability” may carry the risk of negatively affecting peri-implant tissue health. Recent animal studies have explored the interplay between mechanical factors and biological responses in osseointegration. These studies have shown that inserting dental implants at higher torque can increase the extent of interfacial microfractures, which are associated with bone resorption and may compromise osseointegration [24,25,26,27,28,29]. Conversely, implants placed with lower insertion torque have been shown to induce smaller compressive strains in peri-implant bone, although this may exacerbate the stability “dip” observed in long-term implant stability curves [24,30,31].

The main challenge to date has been that, for most implant modifications, there has been a clear relationship between higher insertion torque and higher primary stability—an association that is not always advantageous, particularly in softer bone.

For this reason, recent dental research has focused on developing implant designs that provide sufficient primary stability in softer bone (e.g., maxillary regions) without adversely affecting osseointegration in denser bone (e.g., mandibular regions). The aim of the present study was to identify an implant geometry modification that would allow a reduction in insertion torque without causing a corresponding decrease in primary stability. This phenomenon was investigated in both high-density and low-density bone models. Specifically, the study compared the insertion torque and resonance frequency analysis (ISQ) values of two implants with identical macrogeometry but differing in the pitch of the coronal 3 mm threads, when placed into low-density (D3) and high-density (D1) polyurethane foam blocks simulating human bone.

## 2. Materials and Methods

### 2.1. Polyurethane Blocks

**Figure 1:**
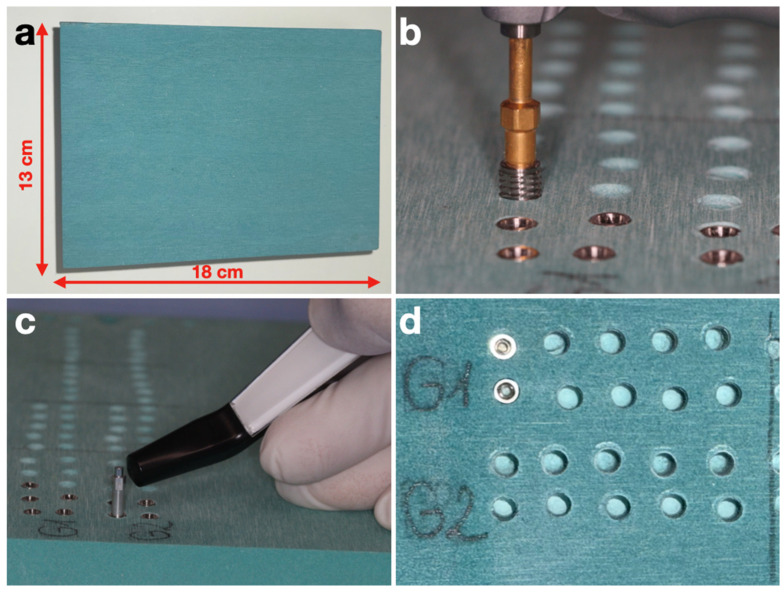
Experimental setup in the high-density polyurethane (PU) block: (**a**) PU block (40 PCF) corresponding to D1-type bone; (**b**) placement of an implant into the block; (**c**) measurement of implant stability using the Osstell Beacon device; (**d**) drill holes and inserted implants in the PU block.

Nowadays, solid rigid polyurethane foam represents the most suitable model to perform in vitro tests and to compare bone screws and dental implants in order to simulate different consistencies and densities of natural bone, as has already been reported [32,33,34]. In this study, 13 cm × 18 cm × 4 cm polyurethane blocks (Nacional Ossos, Jaú, SP, Brazil) with 20 and 40 PCF in density (which correspond to the in vivo D3 and D1 bone, respectively, according to Misch classification [35,36,37,38]) were used to test the implants (Figure 1). The microstructure of these materials has also been previously analyzed [33,39].

### 2.2. Implants

**Figure 2:**
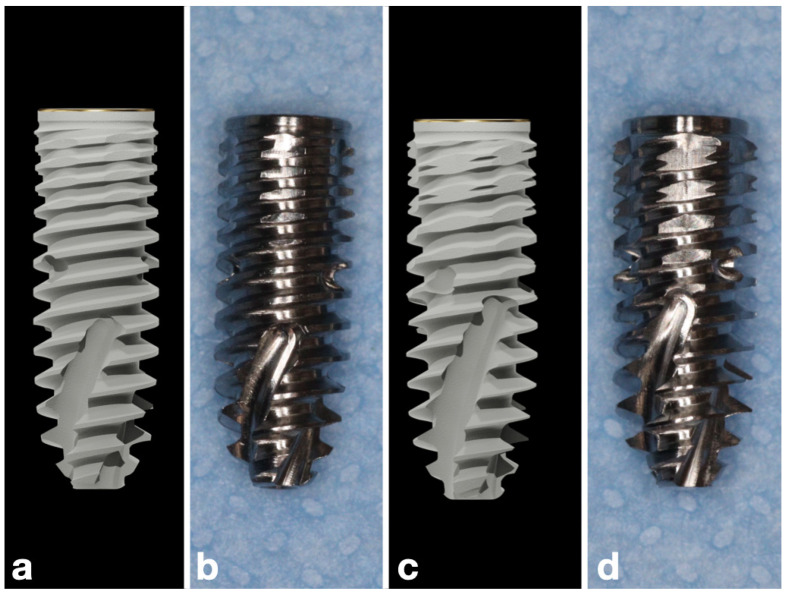
Dental implants used in the study: (**a**) Computer-generated 3D model of the test implant modified P5D design with increased coronal thread density; Group 1; (**b**) photograph of the test implant; (**c**) computer-generated 3D model of the control implant; Group 2; (**d**) photograph of the control implant standard P5D design with regular coronal thread pitch.

Two variants of the P5D conical implant (SGS Dental Implant System Holding AG, Zug, Switzerland) were investigated in this study. Both variants shared identical overall dimensions (4.2 mm diameter, 11.5 mm length), taper, apical configuration, and surface treatment. The only design difference concerned the coronal 3 mm of the implant body, where the thread geometry was modified. Accordingly, the implants were divided into two groups: Group 1 (modified P5D design with increased coronal thread density in the coronal 3 mm standard P5D design with regular coronal thread pitch) and Group 2 (control—standard P5D design with regular coronal thread pitch) (Figure 2).

Implants in Group 1 were characterized by a 27% higher number of threads in the coronal 3 mm compared with the standard design (control—Group 2). This denser threading resulted in a finer pitch and a double-lead-like configuration at the neck, while maintaining identical thread depth and angle. The modification increased the functional surface area and provided tighter engagement with cortical bone. From a biomechanical perspective, this configuration was intended to improve crestal stability by distributing occlusal loads more evenly, enhancing bone-to-implant contact, and reducing micromotion at the cortical level.

Implants in Group 2 featured the original P5D microgeometry (SGS Dental Implant System Holding AG, Zug, Switzerland), characterized by a uniform single-lead thread pitch extending from apex to neck. The coronal region maintained the same pitch and thread depth as the mid-body, resulting in a continuous thread profile designed to provide balanced insertion properties across different bone densities.

Both implant variants shared the same moderately rough surface, achieved by grit blasting and acid etching, to promote osseointegration. A total of 30 implants of each variant were inserted into both 20 and 40 PCF polyurethane foam blocks, resulting in 120 site preparations overall.

### 2.3. Protocol and Stability Testing

The following drilling protocols were used (as recommended by the manufacturer according to bone density): For D1 (high density), preparation began with a pointed initial bur, followed by sequential drilling with 600 rpm, a Ø 1.5 mm bur, a Ø 2.0 mm bur, a Ø 2.5 mm bur, a Ø 2.8 mm bur, a Ø 3.2 mm bur, a Ø 3.7 mm bur and finally a Ø 4.0 mm bur. For D3 (low density), preparation likewise began with a pointed initial bur, followed by sequential drilling with 600 rpm a Ø 1.5 mm bur, a Ø 2.0 mm bur, a Ø 2.5 mm bur, a Ø 2.8 mm bur and a Ø 3.2 mm bur. Although the manufacturer’s protocol recommends a broader drilling sequence, in this study we used drilling at 600 rpm without irrigation, as advised for procedures in polyurethane blocks [40]. The final insertion of the implants was conducted at 30 rpm with a calibrated maximum torque of 80 N·cm [41,42,43]. An Osstell Beacon device (Osstell, Gothenburg, Sweden) was used to obtain the ISQ values. Measurements were performed in two directions in all cases, bucco-lingual (BL) and mesio-distal (MD), according to the manufacturer’s instructions [9,44,45].

Implants were assigned to the experimental groups in equal numbers for each bone density. To minimize potential sequence and handling effects, the order of implant placement was alternated between implant designs throughout the experimental sessions. All implant insertions were performed by the same operator using a standardized drilling and insertion protocol.

Blinding of the operator was not feasible due to visible differences in implant geometry; however, insertion torque values were recorded automatically by the calibrated surgical motor, and implant stability measurements were obtained using resonance frequency analysis according to the manufacturer’s instructions, thereby minimizing operator-dependent measurement bias.

### 2.4. Statistical Analysis

Statistical analyses were performed using Jamovi 2.3.21 (The Jamovi Project) and SPSS 26.0 (IBM, Armonk, NY, USA). Descriptive statistics were calculated for all variables. Categorical variables were reported as absolute frequencies and percentages, while continuous variables were summarized using means, medians, standard deviations, 95% confidence intervals, and observed ranges. Normality was assessed using the Shapiro–Wilk test. As most variables deviated from normality, comparisons between Group 1 and Group 2 were conducted using the non-parametric Kruskal–Wallis H test.

Additionally, in the high-density bone group (D1), a generalized linear model (GLM) was used to assess the independent effects of insertion torque and implant group on primary stability (ISQ mean). The model included torque as a continuous predictor and group (1 vs. 2) as a categorical variable. Regression coefficients, standard errors, 95% confidence intervals, and significance levels were reported. GLM analysis was not performed in the low-density bone group (D3) due to the absence of significant differences in ISQ values. In the D1 group, a generalized linear model (GLM) with a Gaussian error distribution and identity link function was used to evaluate the independent effects of insertion torque (continuous predictor) and implant design (categorical predictor) on mean ISQ. This specification was selected because mean ISQ represents a continuous outcome and the identity link provides directly interpretable regression coefficients.

Because this was an exploratory in vitro study using standardized polyurethane bone analogues and a fixed sample size commonly applied in comparable biomechanical investigations, no a priori sample size or power calculation was performed. The sample size was selected to allow balanced comparisons between implant designs across bone density groups. A sensitivity analysis based on the observed variance of insertion torque indicated that, with 30 implants per group and a two-sided α level of 0.05, the study had >99% power to detect the between-group differences observed in both D1 and D3 bone models. For descriptive purposes, both mean and median values are reported; however, medians are emphasized for non-parametric group comparisons, whereas mean ISQ values are reported when referring to regression-based analyses, as the GLM estimates conditional means.

## 3. Results

### 3.1. Descriptive Statistics and Normality Testing

A total of 120 implants were inserted into rigid polyurethane blocks, simulating two different bone densities. Sixty implants were placed in high-density blocks (40 PCF, corresponding to D1 bone), and sixty in low-density blocks (20 PCF, corresponding to D3 bone). Within each bone density, thirty implants belonged to Group 1, which featured an increased number of coronal threads while maintaining identical dimensions and geometry as the control group and thirty to Group 2, which used the original implant design.

Descriptive statistics for insertion torque and implant stability values, measured via resonance frequency analysis (ISQ) in bucco-lingual (BL) and mesio-distal (MD) directions, as well as their mean values, are summarized in Table 1. The distribution of the data was assessed using the Shapiro–Wilk test. Most variables deviated significantly from a normal distribution (*p* < 0.05), and non-parametric tests were therefore applied in subsequent analyses.

### 3.2. High-Density Bone (D1)

In the D1 bone analogues, Group 2 implants demonstrated significantly higher insertion torque values compared to Group 1. The median insertion torque was 74.0 N·cm in Group 2 and 63.5 N·cm in Group 1. This difference was statistically significant, as shown by the Kruskal–Wallis test (χ^2^ = 16.45, df = 1, *p* < 0.001), and the effect size was substantial (ε^2^ = 0.2788).

Implant stability, measured as ISQ values, was also significantly higher in Group 2 across all measurement directions. The difference in bucco-lingual ISQ values reached statistical significance (χ^2^ = 4.71, *p* = 0.030), as did the mesio-distal values (χ^2^ = 7.56, *p* = 0.006). The average ISQ value was likewise significantly greater in Group 2 than in Group 1 (χ^2^ = 5.82, *p* = 0.016). Effect sizes (ε^2^) for these comparisons ranged from 0.0798 to 0.1281, indicating a moderate impact of the thread modification on implant stability in dense bone.

**Figure 3:**
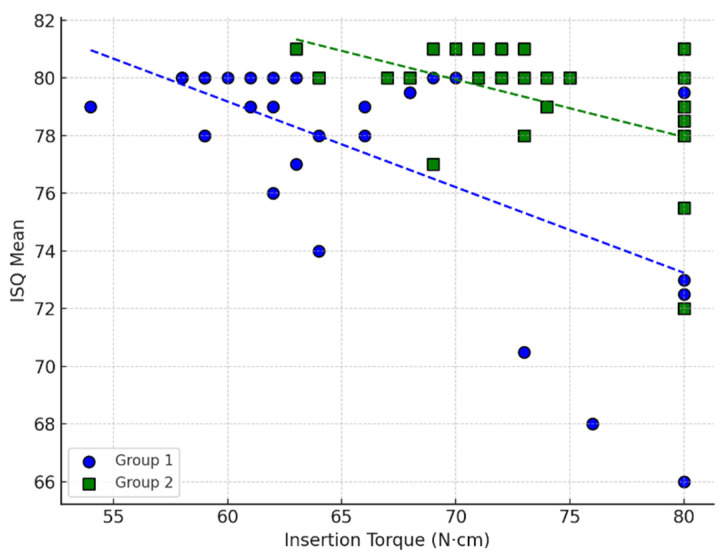
Scatterplot showing the relationship between insertion torque (N·cm) and implant stability (ISQ mean) for Group 1 (implant with increased coronal thread density) and Group 2 (original implant design—control) in high-density polyurethane blocks (D1). Each point represents one implant. Dashed lines indicate linear regression fits for each group.

To further examine the relationship between insertion torque and implant stability, a generalized linear model was constructed using the ISQ mean as the dependent variable. The model identified insertion torque as a significant negative predictor of ISQ, with an unstandardized regression coefficient of −0.267 (SE = 0.0516, t = −5.17, *p* < 0.001). This indicates that for each 1 N·cm increase in insertion torque, the ISQ value decreased by approximately 0.27 points. Additionally, group membership was a significant predictor, with Group 2 associated with a mean ISQ increase of 3.90 points (standard error = 0.8005, t = 4.87, *p* < 0.001) relative to Group 1. These results suggest that although lower torque is generally associated with slightly higher ISQ, this relative advantage was insufficient for the modified coronal-thread design to surpass the primary stability achieved by the original implant in dense bone. A graphical summary of this regression model is presented in Figure 3.

### 3.3. Low-Density Bone (D3)

In the D3 group, the original implants (Group 2) again showed significantly higher insertion torque values than the modified design (Group 1), with median values of 37.5 N·cm and 33.0 N·cm, respectively. This difference was statistically significant (χ^2^ = 33.324, *p* < 0.001), and the effect size was large (ε^2^ = 0.5648).

However, in contrast to the results observed in D1 bone, the ISQ values did not significantly differ between groups. No statistically significant difference was found in bucco-lingual ISQ values (χ^2^ = 0.844, *p* = 0.358), mesio-distal values (χ^2^ = 0.805, *p* = 0.370), or mean ISQ (χ^2^ = 0.655, *p* = 0.418). All associated effect sizes were negligible (ε^2^ < 0.015). Due to the absence of a significant difference in stability outcomes, regression analysis was not conducted for the D3 group, as the assumptions and interpretability of the model were not met.

## 4. Discussion

In the present in vitro study, modification of the coronal thread pitch significantly influenced insertion torque and primary stability in a bone-density-dependent manner. In high-density (D1) bone, increasing coronal thread density resulted in a significant reduction in insertion torque, accompanied by a moderate but statistically significant decrease in ISQ values. In contrast, in low-density (D3) bone, the reduced coronal thread pitch lowered insertion torque without causing a corresponding reduction in primary stability, as reflected by comparable ISQ values between implant designs. Importantly, in both bone qualities, ISQ values consistently remained above clinically accepted thresholds for immediate loading.

In the scientific literature, it is already reported that the macroscopic structure and the surface characteristics of dental implants play a decisive role in obtaining success in osseointegrated implantology [46]. In particular, the geometrical design of the threads, their position, and their pitch along the implant body determine different responses to functional loads and the transmission of those forces to the surrounding bone tissue [47]. The implant design plays an even more important role if surgical protocols providing immediate loading are adopted. It is known that in the initial stages following implant insertion, and especially after immediate loading, implant stability should be guaranteed by the mechanical relationship between the fixture and the bone tissue rather than biological bone integration. Therefore, the percentage of BIC and the friction obtained during insertion play an important role in the mechanical behavior of immediately loaded prosthetic implants.

The tapered shape of the implant fixtures ensures a gradual expansion of the thin crests during the insertion phase of the fixture, producing the least possible stress on the surrounding bone. This factor is of fundamental importance in cases of reduced bone availability, where preserving cortical bone tissue is appropriate, as well as carrying out a three-dimensional expansion and compaction of the walls of the newly formed alveolar bone.

In this study, reducing the coronal thread pitch (increasing the coronal thread density) of the implant significantly reduced the insertion torque in dense (D1) bone, which is in itself a favorable outcome. In the commercially available implant used as the control, the measured torque values often reached 80 N·cm, with a mean exceeding 74 N·cm; however, our results showed that this control implant also exhibited significantly higher primary stability. In contrast, the implants with the modified (reduced) thread pitch demonstrated more moderate insertion torque values (mean of 66 N·cm) and reached the critical threshold of 80 N·cm less frequently—occurring in 16.7% of implants in the test group compared with 36.7% in the control group. Although this difference showed a clear trend, it was not statistically significant. The primary stability values were also significantly lower for the modified implants compared with the control group, which is a less favorable outcome. It should be noted, however, that even these lower primary stability values were well above the levels recommended in the literature for immediate loading, and in no case did the primary stability drop below 35 N·cm, which is generally considered more than sufficient for immediate loading [48,49].

The implant type analyzed showed a thread design that releases less force but still allows easy access to good primary stability. The thread geometry contributes to obtaining primary stability, which is responsible for the biomechanical behavior of the bone–implant interface after the healing process [50].

Thread height is defined as the distance between the major and the minor diameter of the coil. A shallow thread depth, favors insertion resulting less force needed for the insertion. In fact, although deeper threads increase the surface area and represent an advantage in areas of low-density bone and high occlusal stress, shallow threads, on the other hand, allow easier insertion in alveolar ridges with denser bone without the need for tapping before implant insertion [51].

In addition, these threads have an osteotomic effect, allowing the peri-implant bone to be compacted using a surgical technique that provides preparation of the implant site according to the “press-fit” protocol. In vitro studies showed that in cases of poor-quality bone, such as in the posterior maxilla, implants with a chamfer thread design produced lateral compressive forces that increased the BIC and consequently improved primary stability [52]. This factor is very important in cases using the immediate load technique with several implants, as in rehabilitation providing immediate solidification using bar techniques. Furthermore, as already demonstrated, under vertical load the presence of threads with a bevel peak allows a reduction of divergent forces, thereby reducing stress at the bone–implant interface [53,54,55].

Implants with greater thread depth have shown higher insertion torque and implant stability quotient (ISQ) values in soft bone models in several studies [47,48]. Finite element analyses also support that optimized thread parameters (depth, width, pitch, and angle) can reduce bone strain and improve primary stability [56,57]. Nevertheless, the issue of primary stability can also have negative effects on implant longevity if the applied forces exceed certain limits. Excessive compression from high torque may exceed the physiological tolerance of the bone, leading to necrosis and early bone loss, particularly in dense bone or in thin cortical layers [25,58]. Excessive insertion torque can also cause surface damage to implant threads, resulting in the release of titanium particles at the bone–implant interface, especially at the crestal bone. This may contribute to marginal bone loss and peri-implant inflammation, although its long-term clinical significance is still under investigation [59,60]. High torque further increases strain and can cause microcracks, plastic deformation, and a larger zone of dead or dying osteocytes in peri-implant bone, potentially leading to increased bone resorption and impaired healing [25,61]. Despite these risks, several studies and meta-analyses have reported no significant difference in marginal bone loss between high (≥50 N·cm) and regular/low torque (<50 N·cm) placements over 1–3 years [62,63,64]. However, some clinical and animal studies have shown greater early bone loss and soft tissue recession with high torque, particularly in thin bone or fresh extraction sites [65,66,67]. Mechanical testing by Gehrke and colleagues demonstrated that torques above 80 N·cm can deform implant structures, while some anti-rotational systems may be compromised at even lower values (36 N·cm) [68]. In the present study, the 80 N·cm limit both protected the implants used from deformation and introduced a bias, since higher torque values could not be reached with the device employed. To avoid implant deformation, no torque higher than 80 N·cm was applied manually.

The present findings should be interpreted in light of several limitations inherent to the experimental design. First, the use of rigid polyurethane foam blocks, although widely accepted for standardized in vitro implant testing, cannot fully reproduce the complex biological and mechanical behavior of human bone, particularly with respect to remodeling and vascular response. Second, the maximum insertion torque was intentionally limited to 80 N·cm to avoid implant deformation; while clinically relevant, this ceiling may have introduced a measurement bias by preventing the assessment of higher torque values that could further differentiate implant designs. Third, the absence of dynamic or cyclic loading precludes evaluation of how the observed primary stability differences would evolve under functional conditions. Finally, bone-to-implant contact (BIC) was not directly measured, and therefore interpretations based on ISQ values reflect mechanical stability only and cannot be extrapolated to histological osseointegration. These limitations should be considered when translating the present in vitro results to clinical practice.

Our results also show that with the drilling protocol used and the selected implant type, sufficient stability was achieved even with the version of the implant featuring a reduced thread pitch in dense bone. Determining the optimal insertion torque and ISQ value is challenging, particularly because a given modification may have different effects in soft versus dense bone. It is already well established that deeper threads generally provide greater primary stability; however, in our study the key question was whether increasing the thread density could facilitate smoother insertion with less force, without compromising primary stability. This goal was achieved in soft bone, but in dense bone the reduction in insertion torque was accompanied by a decrease in primary stability. Nevertheless, it should be emphasized that in both dense and soft bone the mean ISQ values consistently remained well above the commonly accepted minimum threshold of 60 [69,70]. Therefore, even with the reduced insertion torque, the achieved ISQ values were sufficient for immediate loading in all cases, regardless of bone quality.

## 5. Conclusions

This in vitro study demonstrated that increasing the density of coronal threads in dental implants significantly decreases primary stability, but its effect is dependent on bone quality. In high-density bone (D1), implants with a greater number of coronal threads achieved somewhat lower insertion torque and lower implant stability (ISQ values), in low-density bone (D3), although insertion torque values were significantly lower for the modified implants, no corresponding decrease in implant stability was observed. Reducing the thread pitch reduces insertion torque, which is a favorable characteristic; however, in dense bone this reduction is accompanied by a decrease in primary stability. Nevertheless, this decrease still yields stability levels sufficient for immediate loading, suggesting that the modification may help prolong implant survival by lowering the likelihood of bone resorption caused by excessive insertion forces. Based on these findings, the reduced thread pitch appears particularly advantageous in softer bone, as the reduced insertion torque causes less damage to the inherently more fragile bone without compromising stability. In dense bone, although a slight reduction in stability can be expected, the measured values remain well above the minimum required thresholds; therefore, when weighed against the benefits of lower insertion torque, the modified design can still be considered to offer improved overall outcomes.

## Figures and Tables

**Table 1:** Descriptive statistics (mean, SD, min–max, median) for insertion torque (N·cm) and ISQ values across groups 1 and 2 in D1 and D3 bone densities.

	**D1—Group 1**					**D1—Group 2**				
	**Mean**	**Median**	**SD**	**Min**	**Max**	**Mean**	**Median**	**SD**	**Min**	**Max**
**Torque**	66	63.5	7.86	54	80	74.2	74	5.24	63	80
**ISQ BL**	77.2	79	4.34	62	80	78.9	80	2.87	70	81
**ISQ MD**	77.6	79	3.82	62	80	79.3	80	2.02	74	81
**ISQ Mean**	77.4	79	3.8	66	80	79.1	80	2.34	72	81
	**D3—Group 1**					**D3—Group 2**				
	**Mean**	**Median**	**SD**	**Min**	**Max**	**Mean**	**Median**	**SD**	**Min**	**Max**
**Torque**	31.9	33	4.35	12	38	37.6	37.5	2.65	33	44
**ISQ BL**	66.4	67	1.61	63	69	65.9	66	1.82	62	68
**ISQ MD**	66.4	66.5	1.71	61	69	66	66	1.73	62	68
**ISQ Mean**	66.4	66.5	1.53	63.5	69	66	66	1.76	62	68

## Data Availability

The analysis dataset is available from the corresponding author on reasonable request.
